# Emotional Burden of Patients With Work‐Related Hand Eczema: Results of an Exploratory Study in a Tertiary Individual Prevention Setting in Germany

**DOI:** 10.1111/cod.14819

**Published:** 2025-06-01

**Authors:** Marc Rocholl, Richard Brans, Annika Wilke, Julia Meyer, Swen Malte John, Michaela Ludewig

**Affiliations:** ^1^ Department of Dermatology, Environmental Medicine and Health Theory, Institute for Health Research and Education Osnabrück University Osnabrück Germany; ^2^ Institute for Interdisciplinary Dermatological Prevention and Rehabilitation (iDerm) Osnabrück University Osnabrück Germany; ^3^ Lower Saxony Institute of Occupational Dermatology (NIB) Osnabrück University Osnabrück Germany; ^4^ Fachbereich Gesundheitswissenschaften Hochschule Bochum Bochum Germany

**Keywords:** cross‐sectional study, disease burden, emotional impact, Germany, work‐related hand eczema

## Abstract

**Background:**

Work‐related hand eczema (WRHE) is a prevalent skin condition associated with reduced health‐related quality of life (HRQoL), sleep disturbances or depression. While prior studies primarily explored emotional effects as part of HRQoL, emotions have been less studied separately.

**Objectives:**

To evaluate the psychometric properties of the ‘Atopic Eczema Score of Emotional Consequences’ (AESEC) in patients with WRHE and to investigate the emotional impact of WRHE.

**Methods:**

Self‐reported sociodemographic data as well as self‐reported and clinically assessed disease severity of 223 patients (55.6% female; mean age: 48.1 ± 12.0 years) taking part in a tertiary prevention program for WRHE were included in the analysis. The factor structure was checked using an exploratory factor analysis. The emotional burden was determined using the AESEC.

**Results:**

The three underlying facets of emotional consequences, as previously described in the validation study of the AESEC, could not be replicated in patients with WRHE. Positive emotions were emphasised by items such as optimism, balance and self‐confidence. Emotional burden was most evident in worries about life, sadness, lack of control, feelings of constraint and disturbance by itch.

**Conclusions:**

Patients with WRHE face a significant emotional burden indicating a great need for psychological support. Further studies on the psychometric properties of the AESEC in patients with WRHE are warranted.

## Graphical abstract

Work‐related hand eczema is a prevalent skin condition that imposes significant emotional burden on patients, including worries about daily life, sadness, lack of control, feelings of constraint and disturbance by itch. This highlights the urgent need for psychological support.

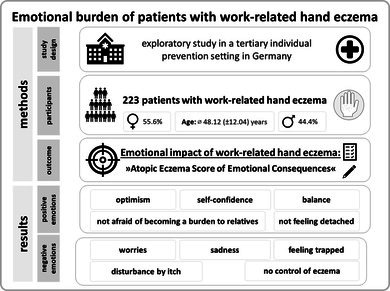

**Abbreviations**
AESEC: Atopic Eczema Score of Emotional Consequences
EAS: Erlangen Atopy Score
HE: hand eczema
HRQoL: health‐related quality of life
KMO: Kaiser–Meyer–Olkin criterion
M: mean
Max: maximum
Md: median
Min: minimum
OHSI: Osnabrueck Hand Eczema Severity Index
POEM: Patient‐Oriented Eczema Measure
QOLHEQ: Quality Of Life in Hand Eczema Questionnaire
SD: standard deviation
TIP: tertiary individual prevention program
WRHE: work‐related hand eczema

## 1 Introduction

Hand eczema (HE) is a very common and often chronic skin disease characterised by inflammatory skin lesions on the hands, with a point‐ and 1‐year prevalence in the general population of 4.0% and 9.1%, respectively [1]. The classification of HE includes both endogenous subtypes (e.g., atopic HE) and exogenous subtypes (e.g., irritant and allergic contact dermatitis), which are caused or aggravated by occupational or non‐occupational factors [2]. Often, HE consists of mixed or overlapping subtypes [2, 3].

Work‐related HE (WRHE) is one of the most frequently reported occupational diseases in various European countries and therefore has a considerable socioeconomic impact [4, 5, 6]. It is significantly more common in occupations at risk, such as construction workers, healthcare workers or hairdressers [7, 8, 9]. For the individuals affected, (WR)HE is often associated with various detrimental consequences, including reduced health‐related quality of life (HRQoL) or reduced ability to work, (itch‐related) sleep disturbances or depression [10, 11, 12, 13, 14]. The emotional impact (e.g., frustration or feelings of shame) of skin diseases, particularly HE, has only been studied to a limited extent: Grant et al. [11] investigated the emotional impact of chronic (atopic, allergic or irritant) HE in a qualitative study and summarised their findings in a conceptual model. They found affected individuals to be impaired in various domains, including emotions (e.g., embarrassment, frustration, or sadness/depression). Rönsch et al. [15] also reported emotional impairment in patients with chronic HE. Further studies have investigated ‘emotions’ (e.g., anxiety, frustration or sadness) in patients with HE—alongside symptoms, functioning and treatment/prevention—using the ‘Quality Of Life in Hand Eczema Questionnaire’ (QOLHEQ) [16, 17]. Depending on the study sample, these studies found slight to moderate, and in one study even strong impairments of HRQoL in the emotions domain [14, 16, 17, 18, 19, 20, 21]. In contrast to the QOLHEQ, in which the impact of emotions is only one sub‐aspect of HRQoL, Arents et al. [22] developed a questionnaire for patients with atopic dermatitis (‘Atopic Eczema Score of Emotional Consequences’ [AESEC]) that exclusively analyses the emotional consequences of this disease in detail. Studies that used the AESEC indicate a substantial emotional disease burden in patients with atopic dermatitis [22, 23]. Even though there is some overlap between patients with atopic dermatitis and HE, these findings are not entirely transferable to individuals with WRHE. A thorough understanding of the emotional consequences is crucial for health care professionals and to ensure comprehensive and patient‐centred care; thus, it is necessary to investigate the emotional impact of WRHE separately. Therefore, the aims of this exploratory study were (1) to evaluate the psychometric properties of the AESEC in patients with WRHE and (2) to investigate the emotional consequences of WRHE.

## 2 Methods

### 2.1 Study Design

From June 2020 to May 2021, we conducted a cross‐sectional study in our department. Details of the study design and the setting of the conducted survey are reported elsewhere [24]. We enrolled patients participating in a three‐week inpatient tertiary individual prevention (TIP) program for WRHE [25, 26] who were at least 18 years of age, had current or previous HE and had given written informed consent to participate in the study. Recruitment was conducted on the first day of the prevention program. Patients with other skin diseases (e.g., psoriasis, foot eczema or tinea manuum), but who did not have HE either currently or in the past, as well as people with insufficient language skills, were excluded from the study. The study was approved by the ethics committee of Osnabrück University (no. 13/2020).

### 2.2 Questionnaire and Scores

All data were collected on the first day of the inpatient programme. A written questionnaire was used to collect all relevant survey data, such as sociodemographic variables (age, sex, occupation, employment status). The occupation groups were categorised based on classifications used in previously published studies [25, 27, 28] to ensure comparability with the cohorts in those studies.

Self‐reported severity of HE was assessed using the ‘Patient‐Oriented Eczema Measure’ (POEM) [29, 30]. Originally developed for patients with atopic dermatitis, the POEM is a brief and user‐friendly questionnaire that effectively measures disease severity from the patient's perspective. It has demonstrated good psychometric properties, including high internal consistency (Cronbach's *α* = 0.88) [29]. The POEM evaluates symptom frequency (e.g., itch, sleep disturbance, cracking) over the past week on a 5‐point scale, ranging from 0 = ‘no days’ to 4 = ‘every day’ with a maximum score of 28 points. Specifically, the POEM scores can be interpreted to reflect the severity of atopic dermatitis as follows: a score of 0–2 indicates a condition that is clear or almost clear; 3–7 corresponds to mild eczema; 8–16 represents moderate eczema; 17–24 signifies severe eczema and 25–28 denotes very severe eczema. For our study purposes, we terminologically rephrased the POEM by using the term ‘hand eczema’ instead of ‘eczema’.

Furthermore, we used a global item with a 5‐point rating scale to assess the disease severity (‘How would you describe the severity of your hand eczema?’: (1) *clear*; (2) *almost clear*; (3) *mild*; (4) *moderate* and (5) *severe*). In addition, a dermatologist assessed the disease severity using the ‘Osnabrueck Hand Eczema Severity Index’ (OHSI) [31, 32] and evaluated the atopic disposition of the study participants using the ‘Erlangen Atopy Score’ (EAS) [33, 34]. The diagnosis of HE and its subtypes was determined by experienced dermatologists at our clinic.

### 2.3 Modified AESEC

Emotional consequences of HE were assessed using the AESEC [22]. The AESEC was developed in an iterative process within an international research project aimed at creating an instrument to measure the burden of disease—in particular the emotional consequences—of people affected by atopic dermatitis. The instrument consists of 28 items and demonstrates good psychometric properties (e.g., high reliability: Cronbach's *α* = 0.900 and low inter‐item correlation: *p* = 0.208). The items of a questionnaire are usually organised in so‐called factors. These represent different facets of an underlying construct that the questionnaire aims to measure. The items of the AESEC can be allocated to the following three factors: desperate/burdened (Cronbach's *α* = 0.882), insecure/worried (Cronbach's *α* = 0.822), and balanced/satisfied (Cronbach's *α* = 0.802). Items are rated on a 4‐point Likert scale (‘applies fully’, ‘somewhat applies’, ‘rather not’, ‘does not apply at all’) resulting in a possible total score ranging between 0 and 84 points, with higher values indicating stronger emotional burden: 0–27: ‘no/small effect’; 28–39: ‘moderate effect’; 40–52: ‘large effect’; 53–84: ‘very large effect’ [22]. As mentioned above, the AESEC was terminologically rephrased for the purposes of our study by using the term ‘hand eczema’ instead of ‘eczema’.

### 2.4 Statistical Analyses

Statistical analyses were carried out using IBM SPSS version 28 (SPSS Inc., Chicago, IL) [35]. Descriptive statistics were calculated for all variables. Means (M), medians (Md) and standard deviations (SD) were calculated for continuous variables (e.g., age, duration of disease). Categorical data (e.g., sex, family status) are presented as frequencies and percentages.

### 2.5 Psychometric Evaluation

Prior to the analysis, missing values were analysed, and the plausibility of the data was checked. Datasets with missing values in the AESEC range of ≥ 5% were excluded; missing values in datasets with < 5% in the AESEC range were imputed using the EM algorithm implemented in SPSS [36, 37]. The structure was checked using an exploratory factor analysis with principal component analysis and VARIMAX rotation. The appropriateness of the variables was tested using the Kaiser–Meyer–Olkin (KMO > 0.50) criterion and the Bartlett test for sphericity. The Kaiser–Guttman criterion (eigenvalue > 1) was used to identify the number of factors. Cronbach's *α* (*α* > 0.70: acceptable; *α* > 0.80: good [38]) was calculated to analyse the internal consistency of the AESEC scales. The quality of the items was assessed based on item difficulty (item mean values), the factorial loading structure (loadings > 0.40) and the corrected discriminatory power (> 0.40) [39]. If the factor loading of an item was divided among several factors, the allocation to a factor was based on contextual criteria.

## 3 Results

Of the 291 patients who were invited to participate in the study, 254 (87.3%) gave informed consent and completed the questionnaire. Of these, 31 were excluded due to a large number (> 5%) of missing values. Finally, 223 datasets were included in the analyses. Sociodemographic characteristics of patients enrolled in the study are presented in Table 1. Table 2 provides details of the disease severity and the atopy screening results. Table 3 presents the diagnoses of the study sample.

**TABLE 1:** Sociodemographic characteristics of the study sample (*N* = 223).

	*N*	%
Sex		
Female	124	55.6
Male	99	44.4
Age (years)		
M ± SD (min–max)	48.1 ± 12.04 (18–66)	
Duration of disease^a^ (years)		
M ± SD (min–max)	8.0 ± 10.3 (0.2–60)	
Md	4.0 years	
Occupation groups		
Healthcare/elderly care workers	78	35.0
Metal workers	10	4.5
Mechanics	33	14.8
Hairdressers, cosmetologists, close contact services	7	3.1
Construction workers	12	5.4
Food handlers	16	7.2
Cleaning personnel	2	0.9
Gardeners/florists	3	1.3
Others	54	24.2
Not specified/prefer not to say	8	3.6
Employment status		
Self‐employed	7	3.1
Employed	211	94.6
Unemployed	4	1.8
Not specified/prefer not to say	1	0.4

Abbreviations: M = mean; Max = maximum; Md = median; min = minimum; SD = standard deviation.

^a^ *N* = 211 (missing values: *N* = 12).

**TABLE 2:** Disease severity and results of atopy screening (*N* = 223).

	*N*	%
OHSI		
M ± SD (min–max)	6.4 ± 3.1 (0–15)	
POEM^a^		
M ± SD (min–max)	14.41 ± 6.6 (0–28)	
Md	14.0	
0–2 (clear or almost clear)	8	3.7
3–7 (mild *hand* eczema)	29	13.3
8–16 (moderate *hand* eczema)	98	45.0
17–24 (severe *hand* eczema)	68	31.2
25–28 (very severe *hand* eczema)	15	6.9
Global self‐assessment		
M ± SD (min–max)	3.9 ± 0.8 (1–5)	
Md	4.0	
Clear	3	1.3
Almost clear	10	4.5
Mild	27	12.1
Moderate	131	58.7
Severe	42	18.8
Not specified/prefer not to say	10	4.5
Erlangen atopy score		
M ± SD (min–max)^b^	8.5 ± 5.0 (0–24)	
Md^b^	8.0	
0–3 (no atopic skin diathesis)	37	16.6
4–7 (improbable atopic skin diathesis)	69	30.9
8–9 (indistinct atopic skin diathesis)	27	12.1
≥ 10 (atopic skin diathesis)	90	40.4

Abbreviations: M = mean; Md = median; OHSI = Osnabrueck Hand Eczema Severity Index; POEM = Patient‐Oriented Eczema Measure; SD = standard deviation.

^a^ *N* = 218 (excluded due to two or more missing values: *N* = 5).

^b^ *N* = 222 (missing value: *N* = 1).

**TABLE 3:** Subtypes of hand eczema in the study sample (*N* = 223, multiple subtypes possible).

Subtypes of hand eczema	*N*	% of all responses^a^
Atopic hand eczema	144	39.2
Irritant contact dermatitis	144	39.2
Allergic contact dermatitis	36	9.8
Hyperkeratotic hand eczema	40	10.9
Not classifiable hand eczema	3	0.8

^a^ The total number of patients who responded is *N* = 223. However, because multiple subtypes of hand eczema can coexist in a single patient, more than one response is possible. The percentages in the right‐hand column of the table refer to the total number of responses, which is 367.

### 3.1 Psychometric Evaluation of the AESEC in Patients With WRHE

Results of the psychometric evaluation (e.g., results of the exploratory factor analysis of the AESEC questionnaire or scale properties) are shown in detail in the additional files (see Tables S1–S3). The previously described three‐factor structure of the AESEC [22] (see Section 2.2) was not replicated in our study sample. The exploratory factor analysis suggests a five‐ to six‐factor structure (see Table S1) with (overall) acceptable to good internal consistency for most of the scales (Cronbach's *α* between 0.683 and 0.875). Descriptive analysis of the items, mean values and discriminatory power are presented Table S2.

### 3.2 Emotional Burden of Patients With WRHE

As the three‐factor structure of the AESEC was not replicated, the items investigating emotional burden are presented only descriptively in Table 4, with the responses grouped into the categories of ‘somewhat applies/applies fully’ and ‘rather not/does not apply at all’. The raw, unbanded options can be found in Table S4. The items that most clearly emphasised the positive emotions of our study participants were as follows: ‘I am optimistic about my life with *hand* eczema’ (AESEC_8; somewhat applies/applies fully: 85.7%); ‘I feel detached from others’ (AESEC_1; rather not/does not apply at all: 80.3%); ‘I'm afraid of being a burden to my relatives’ (AESEC_27; rather not/does not apply at all: 76.6%); ‘I am a well‐rounded person’ (AESEC_14; somewhat applies/applies fully: 75.3%) as well as ‘I am self‐confident’ (AESEC_3; somewhat applies/applies fully: 73.5%).

**TABLE 4:** Descriptive results of the items (grouped responses, not‐imputed dataset; *N* = 223).

Item‐No.	Item	Applies fully, % (*N*)	Somewhat applies, % (*N*)	Rather not, % (*N*)	Does not apply at all, % (*N*)	No answer/missing, % (*N*)
1	I feel detached from others.	19.2 (43)		80.3 (179)		0.4 (1)
2	I try to hide my *hand* eczema.	62.4 (139)		37.2 (83)		0.4 (1)
3	I am self‐confident.	73.5 (164)		26 (58)		0.4 (1)
4	I am nervous.	61.9 (138)		38.1 (85)		—
5	I envy people with normal skin.	65.5 (146)		34.5 (77)		—
6	I feel I can handle my *hand* eczema.	36.7 (32)		62.8 (140)		0.4 (1)
7	I feel overwhelmed by my *hand* eczema.	52.4 (117)		47.5 (106)		—
8	I am optimistic about my life with *hand* eczema.	85.7 (191)		13.9 (31)		0.4 (1)
9	I try to avoid physical contact or touching other people.	51.1 (114)		48.8 (109)		—
10	I struggle with my appearance.	42.1 (94)		57.4 (128)		0.4 (1)
11	I am a relaxed person.	68.6 (153)		30.9 (69)		0.4 (1)
12	My *hand* eczema makes me angry.	54.7 (122)		45.3 (101)		—
13	I'm afraid of being rejected.	28.2 (63)		71.3 (159)		0.4 (1)
14	I am a well‐rounded person.	75.3 (168)		24.2 (54)		0.4 (1)
15	I feel guilty about scratching.	32.3 (72)		67.3 (150)		0.4 (1)
16	I have difficulties concentrating.	39.4 (88)		59.2 (132)		1.3 (3)
17	I am in control of my *hand* eczema.	26.0 (58)		73.5 (164)		0.4 (1)
18	I feel sad about having *hand* eczema.	72.2 (161)		27.8 (62)		—
19	I feel I can do what other people can do.	56.1 (125)		43.9 (98)		—
20	I worry about my life because of my *hand* eczema.	81.2 (181)		18.8 (42)		—
21	I feel embarrassed about my skin appearance.	50.6 (113)		49.3 (110)		—
22	I have no problem with intimacy.	59.6 (133)		39.5 (88)		0.9 (2)
23	I feel insecure.	42.6 (95)		53.3 (119)		4.0 (9)
24	I cope well with my *hand* eczema.	59.2 (132)		40.8 (91)		—
25	I feel trapped because of my *hand* eczema.	73.5 (164)		26.5 (59)		—
26	I feel I am good enough as a person.	70.4 (157)		28.2 (63)		1.3 (3)
27	I'm afraid of being a burden to my relatives.	22.8 (51)		76.6 (171)		0.4 (1)
28	Itching drives me crazy.	72.6 (162)		26.1 (58)		1.3 (3)

The items most likely to represent an emotional burden on the participants were: ‘I worry about my life because of my *hand* eczema’ (AESEC_20; somewhat applies/applies fully: 81.2%); ‘I am in control of my *hand* eczema’ (AESEC_17; rather not/does not apply at all: 73.5%); ‘I feel trapped because of my *hand* eczema’ (AESEC_25; somewhat applies/applies fully: 73.5%); ‘Itching drives me crazy’ (AESEC_28; somewhat applies/applies fully: 72.6%) as well as ‘I feel sad about having *hand* eczema’ (AESEC_18; somewhat applies/applies fully: 72.2%).

## 4 Discussion

To the best of our knowledge, this is the first study evaluating the psychometric properties of the AESEC in patients with WRHE and the first to investigate its emotional impact separately.

We were unable to replicate the three‐factor structure of the AESEC (see Section 2) described in patients with atopic dermatitis [22] in patients with WRHE. Instead, the exploratory factor analysis resulted in a five‐ to six‐factor structure, with acceptable to good internal consistency for most of the identified factors, but also with several cross‐loadings. The items of the factor ‘balanced/satisfied’ described by Arents et al. [22] were divided into two factors in our exploratory factor analysis (Factor 2 ‘balance’ and Factor 5 ‘manageability’—see Table S1). The items of the original factors ‘desperate/burdened’ and ‘insecure/worried’ [22] are distributed almost evenly across the other three factors in our exploratory factor analysis and do not allow clear conclusions regarding their underlying concepts in our study population. While cross‐loadings > 0.4 are occasionally apparent in the work of Arents et al. [22] (e.g., Items No. 10, No. 21 and No. 25), our exploratory factor analysis does not show a factor structure comparable to the clear results reported previously. There are various possible reasons for this: First, our study sample is—in comparison to the sample of Arents et al. [22]—more heterogeneous with regard to the underlying skin condition. While the original instrument was validated in a sample of patients with atopic dermatitis, the aetiology of WRHE is usually more complex, consisting not only of various morphological HE subtypes, but often of mixed and overlapping etiologies [2, 3, 25]. Therefore, items describing a concept applicable to patients with atopic dermatitis may not be equally relevant for patients with (WR)HE. Against the background of the non‐reproducible factor structure, no conclusions regarding emotional consequences—beyond the descriptive analyses of the items presented above—can be drawn. This has to be considered as a limitation of the present study.

In terms of the emotional burden due to itching and feelings of sadness, our findings are similar to those of the validation study [22] and are consistent with the results from other studies investigating the disease burden of chronic HE [11, 15, 40]. In comparison to the results of Arents et al. [22], however, our findings indicate a somewhat diametrically opposed higher emotional burden for some items, for example, item AESEC_6 ‘I feel I can handle my eczema’ (Arents et al. [22]: somewhat applies/applies fully: 77%), AESEC_17 ‘I am in control of my eczema’ (Arents et al. [22]: somewhat applies/applies fully: 75%), AESEC_20 ‘I worry about my life because of my eczema’ (Arents et al. [22]: rather not/does not apply at all: 71%) and AESEC_25 ‘I feel trapped because of my eczema’ (Arents et al. [22]: rather not/does not apply at all: 71%). The higher emotional burden associated with WRHE in our study sample might be related to a higher disease burden in our patient sample, as the TIP is primarily offered to patients with severe, long‐lasting WRHE. In addition, the localisation of the skin disease on the hands might have an important impact, as the hands, for example, in comparison to the forearms or legs, are more visible to others, which may lead to stigmatisation and (therefore) greater emotional burden [15, 41, 42].

A strikingly higher proportion of our study participants expressed ‘worries’ about their WRHE (AESEC_20) compared to what has been reported for patients with atopic dermatitis [22]. This is in line with previous research in patients with chronic HE [15]. Worries related to WRHE could be due to impending economic disadvantages caused by prolonged incapacity to work and reduced earning capacity. Moreover, severe WRHE may lead to unemployment or a change to a precarious occupation, and thus, personal and financial insecurities. However, even if the occupational capacity is maintained, a loss of productivity as a result of HE (e.g., due to avoidance of certain work activities) can be equally burdensome for those affected [11].

### 4.1 Limitations

When interpreting the results of this study, several limitations should be considered. Both the AESEC and the POEM are instruments intended for patients with atopic dermatitis and have been validated only in this patient group and not in (WR)HE patients. Even though atopic dermatitis and WRHE represent different conditions, both are, however, eczematous skin diseases which share common disease signs and symptoms and related consequences, including soreness or itching, sleep disturbances, feelings of stigmatisation and reduced quality of life [42]. Moreover, atopic dermatitis is a significant risk factor for HE, and patients with atopic dermatitis frequently experience (atopic) HE [1, 43]. In addition, the impaired skin barrier in atopic dermatitis facilitates the development of irritant contact dermatitis, which is the main cause of WRHE. In line with this, a high share of TIP patients suffers from atopic dermatitis. Mixed and overlapping aetiologies of HE are very common in TIP patients with WRHE [25], frequently involving atopic HE, which is also evident in our sample. Therefore, both instruments were applied in this exploratory study. The current findings may, however, indicate only a limited fit of the AESEC and its underlying constructs in TIP patients with WRHE. Further research is needed to investigate whether the development of a new or adapted instrument for assessing the emotional consequences of (WR)HE is required. In addition, it is important to bear in mind when interpreting the results of this study that the data was collected on the first day of the TIP, thus before the onset of intensified therapies and psychological support which are provided as part of the program. It is unclear how persistent the emotional burden is in the investigated cohort over time in general and in the course of the prevention program.

It is furthermore essential to emphasise that patients participating in the TIP often have severe, long‐lasting, and refractory HE, which usually results in a particularly high burden of disease. Therefore, the results of our study are limited to the investigated patient group and cannot be considered transferable to other patient populations with (WR)HE. Another limitation of the presented explorative study is the lack of using patient‐reported outcome measures validated for WRHE. Future studies should include patient‐reported outcome measures to enhance the understanding of this burden and to validate the modified AESEC for patients with WRHE, particularly in terms of concept and content validation. Besides this, the cross‐sectional design of our study is a limitation as no conclusions can be drawn as to whether and how the emotional burdens change, for example, over the period of the TIP. Future studies should be conducted with a longitudinal design and investigate the emotional burden of patients with WRHE over a longer period of time with multiple measurement points. The particular strength of the present study lies in the first‐time use of the AESEC in patients with WRHE, which enables in‐depth investigation of its emotional consequences.

## 5 Conclusions

In conclusion, our analysis highlights the significant emotional burden faced by patients with WRHE taking part in an inpatient TIP program indicating a strong need for tailored psychological support. However, considering the limitations of the presented exploratory study, further research on the validity of the underlying constructs and the psychometric properties of the AESEC, including further studies in patients with WRHE, is warranted.

## Author Contributions

**Marc Rocholl:** conceptualization, methodology, software, formal analysis, investigation, data curation, writing – original draft, writing – review and editing, visualization, project administration, funding acquisition. **Richard Brans:** formal analysis, writing – original draft, writing – review and editing, supervision. **Annika Wilke:** conceptualization, methodology, writing – review and editing, supervision, project administration, funding acquisition. **Julia Meyer:** methodology, writing – review and editing, funding acquisition. **Swen Malte John:** writing – review and editing, supervision, funding acquisition. **Michaela Ludewig:** conceptualization, methodology, software, formal analysis, investigation, data curation, writing – original draft, writing – review and editing, visualization, funding acquisition.

## Ethics Statement

This study was approved by the ethics committee of the Osnabrück University (13/2020).

## Conflicts of Interest

M.R., R.B., A.W., J.M. and S.M.J. are and M.L. was professionally involved in the education, counselling or medical care of patients with work‐related skin diseases. The authors declare that they have no further conflicts of interest.

## Supporting information


**Table S1.** Results of the exploratory factor analysis of the AESEC questionnaire, scale properties and explanations for item assignment to scales.
**Table S2**. Descriptive analysis of the items (*N* = 223).
**Table S3**. Intercorrelations of the scales.
**Table S4**. Descriptive results of the items (raw, unbanded data; not‐imputed dataset, *N* = 223).

## Data Availability

The data that support the findings of this study are available from the corresponding author upon reasonable request.
