# Supplementary material for: Emotional Burden of Patients With Work‐Related Hand Eczema: Results of an Exploratory Study in a Tertiary Individual Prevention Setting in Germany
Source: Contact Dermatitis. 2025 Jun 1;93(3):243–51. doi: 10.1111/cod.14819 (PMC12318908; doi:10.1111/cod.14819)
Supplement: Supplementary file 1 — Table S1. Results of the exploratory factor analysis of the AESEC questionnaire, scale properties and explanations for item assignment to scales. Table S2. Descriptive analysis of the items (N = 223). Table S3. Intercorrelations of the scales. Table S4. Descriptive results of the items (raw, unbanded data; not‐imputed dataset, N = 223). [file COD-93-243-s001.docx]

Additional files

# Table of contents

| **Supplemental table 1** | Results of the exploratory factor analysis of the AESEC questionnaire, scale properties and explanations for item assignment to scales. | 2 |
| --- | --- | --- |
| **Supplemental table 2** | Descriptive analysis of the items (N=223) | 4 |
| **Supplemental table 3** | Intercorrelations of the scales. | 5 |
| **Supplemental table 4** | Descriptive results of the items, (raw, unbanded data; not-imputed data set, N = 223). | 5 |

# Supplemental table 1

**Title:** Results of the exploratory factor analysis of the AESEC questionnaire, scale properties and explanations for item assignment to scales.

**Additional information:** Cross-loadings >.4 are highlighted in red.

|  | **1** | **2** | **3** | **4** | **5** | **~~6~~** | **Reasons for item allocation** | |
| --- | --- | --- | --- | --- | --- | --- | --- | --- |
| **Cronbach’s alpha** | 0.875 | 0,782 | 0,728 | 0,723 | 0,683 | - |  | |
| **Eigenvalue** | 8.777 | 2.459 | 1.974 | 1.301 | 1.051 | - |  | |
| **% of variance accounted for** | 31.345 | 8.781 | 7.051 | 4.647 | 3.754 | - |  | |
| AESEC_21 ‘I feel embarrassed about my skin appearance.’ | .772 | .250 | .199 | .191 | .124 | .029 | - | |
| AESEC_2 ‘I try to hide my hand eczema.’ | .690 | .054 | .247 | .147 | .178 | .003 | - | |
| AESEC_5 ‘I envy people with normal skin.’ | .617 | -.052 | .122 | .336 | .027 | -.013 | - | |
| AESEC_27 ‘I’m afraid of being a burden to my relatives.’ | .525 | .298 | .140 | .044 | .081 | .124 | - | |
| AESEC_23 ‘I feel insecure.’ | .522 | .398 | .424 | .122 | .026 | .058 |  | |
| AESEC_10 ‘I struggle with my appearance.’ | .502 | .402 | .274 | .122 | .002 | .171 | - | |
| AESEC_1 ‘I feel detached from others.’ | .484 | .375 | .214 | .164 | .113 | .157 | - | |
| AESEC_14 ‘I am a well-rounded person.’ | .035 | .796 | -.084 | .135 | .047 | -.026 | - | |
| AESEC_11 ‘I am a relaxed person.’ | .138 | .773 | .000 | .124 | -.004 | -.042 | - | |
| AESEC_26 ‘I feel I am good enough as a person.’ | .079 | .647 | .275 | -.031 | .341 | .066 | - | |
| AESEC_8 ‘I am optimistic about my life with hand eczema.’ | .161 | .583 | .133 | -.094 | .249 | .306 | - | |
| AESEC_3 ‘I am self-confident.’ | .406 | .527 | .081 | -.215 | .005 | .182 | ~~-~~ | |
| AESEC_13 ‘I’m afraid of being rejected.’ | .471 | .491 | .431 | .167 | .144 | .027 | The item is assigned to factor 1 because “fear of rejection” best fits in with the content of the other items (e.g. shame or envy) of factor 1. | |
| AESEC_15 ‘I feel guilty about scratching.’ | .251 | .176 | .736 | .038 | .073 | -.087 |  | |
| AESEC_28 ‘Itching drives me crazy.’ | .333 | -.165 | .610 | .048 | .222 | .015 | - | |
| AESEC_16 ‘I have difficulties concentrating.’ | .090 | .395 | .524 | .210 | -.216 | .208 | - | |
| AESEC_12 ‘My hand eczema makes me angry.’ | .283 | -.003 | .498 | .362 | .246 | .076 | - | |
| AESEC_4 ‘I am nervous.’ | .274 | .437 | .494 | .152 | -.334 | .016 | - | |
| AESEC_25 ‘I feel trapped because of my hand eczema.’ | .149 | .073 | .057 | .758 | .153 | .124 | - | |
| AESEC_18 ‘I feel sad about having hand eczema.’ | .444 | .040 | .216 | .581 | .035 | -.022 | - | |
| AESEC_19 ‘I feel I can do what other people can do.’ | .051 | .320 | -.057 | .576 | .414 | .132 | Despite the fact that item AESEC_19 also substantially loads on factor 4. we assigned it to factor 5 because the item fits well with the aspect “manageability”. | |
| AESEC_20 ‘I worry about my life because of my hand eczema.’ | .204 | -.045 | .460 | .562 | .127 | -.005 | - | |
| AESEC_7 ‘I feel overwhelmed by my hand eczema.’ | .389 | .047 | .137 | .434 | .052 | .282 | - | |
| AESEC_17 ‘I am in control of my hand eczema.’ | -.048 | .036 | .211 | .103 | .686 | .197 | - | |
| AESEC_24 ‘I cope well with my hand eczema.’ | .304 | .385 | -.162 | .124 | .637 | -.009 | -- | |
| AESEC_6 ‘I feel I can handle my hand eczema.’ | .223 | .062 | .085 | .243 | .627 | -.053 | - | |
| ~~AESEC_22 ‘I have no problem with intimacy.’~~ | -.006 | .064 | -.056 | .072 | .145 | .819 | Two items cannot constitute an independent factor: Item AESEC_9 was therefore assigned to factor 1. As item AESEC_22 has no further substantial cross-loadings and its content is of negligible relevance for the investigated study population. the item was removed. | |
| AESEC_9 ‘I try to avoid physical contact or touching other people.’ | .440 | .133 | .099 | .279 | -.081 | .599 | See above – AESEC_22. | |
| **Additional statistical information:**  Extraction method: Principal component analysis. Rotation method: Varimax with Kaiser normalisation. | | | | | | | |  |

# Supplemental table 2

**Title:** Descriptive analysis of the items (N=223).

**Additional information:** Supplemental table 2 lists commonality, item difficulty (item mean values) as well as the corrected discriminatory power (>0.40). The item discriminatory power was above the cut-off level r_it_ >0.40 for all considered items. This meant that these items predicted acceptable item differentiation.

| **items** | **commonality** | **arithmetic mean** | **corrected item discriminatory power** |
| --- | --- | --- | --- |
| AESEC_21 ‘I feel embarrassed about my skin appearance.’ | .751 | 1.52 (1.00) | .778 |
| AESEC_2 ‘I try to hide my hand eczema.’ | .593 | 1.62 (1.03) | .598 |
| AESEC_5 ‘I envy people with normal skin.’ | .512 | 1.88 (1.09) | .494 |
| AESEC_27 ‘I’m afraid of being a burden to my relatives.’ | .408 | 0.83 (0.94) | .516 |
| AESEC_23 ‘I feel insecure.’ | .629 | 1.30 (0.89) | .693 |
| AESEC_10 ‘I struggle with my appearance.’ | .533 | 1.28 (0.94) | .653 |
| AESEC_1 ‘I feel detached from others.’ | .486 | 0.79 (0.80) | .620 |
| AESEC_14 ‘I am a well-rounded person.’ | .664 | 1.14 (0.75) | .778 |
| AESEC_11 ‘I am a relaxed person.’ | .634 | 1.16 (0.83) | .569 |
| AESEC_26 ‘I feel I am good enough as a person.’ | .622 | 1.14 (0.81) | .617 |
| AESEC_8 ‘I am optimistic about my life with hand eczema.’ | .547 | 0.75 (0.72) | .591 |
| AESEC_3 ‘I am self-confident.’ | .529 | 1.02 (0.86) | .538 |
| AESEC_13 ‘I’m afraid of being rejected.’ | .698 | 1.09 (0.92) | .717 |
| AESEC_15 ‘I feel guilty about scratching.’ | .650 | 1.04 (0.97) | .591 |
| AESEC_28 ‘Itching drives me crazy.’ | .563 | 1.95 (0.98) | .468 |
| AESEC_16 ‘I have difficulties concentrating.’ | .573 | 1.21 (0.90) | .458 |
| AESEC_12 ‘My hand eczema makes me angry.’ | .526 | 1.49 (0.97) | .435 |
| AESEC_4 ‘I am nervous.’ | .645 | 1.63 (0.93) | .491 |
| AESEC_25 ‘I feel trapped because of my hand eczema.’ | .645 | 1.86 (0.88) | .522 |
| AESEC_18 ‘I feel sad about having hand eczema.’ | .585 | 1.87 (0.95) | .583 |
| AESEC_19 ‘I feel I can do what other people can do.’ | .629 | 2.07 (0.84) | .515 |
| AESEC_20 ‘I worry about my life because of my hand eczema.’ | .587 | 1.53 (0.92) | .432 |
| AESEC_7 ‘I feel overwhelmed by my hand eczema.’ | .443 | 1.43 (0.93) | .458 |
| AESEC_17 ‘I am in control of my hand eczema.’ | .568 | 1.95 (0.73) | .410 |
| AESEC_24 ‘I cope well with my hand eczema.’ | .689 | 1.41 (0.72) | .569 |
| AESEC_6 ‘I feel I can handle my hand eczema.’ | .515 | 1.80 (0.79) | .448 |
| ~~AESEC_22 ‘I have no problem with intimacy.’~~ | ~~.704~~ | ~~1.25 (1.05)~~ | - |
| AESEC_9 ‘I try to avoid physical contact or touching other people.’ | .664 | 1.49 (0.97) | .511 |

# Supplemental table 3

**Title:** Intercorrelations of the scales.

**Additional information:** In order to determine intercorrelations between the scales of the questionnaire. Spearman's correlation coefficients were analysed as a measure of construct validity. The correlations indicate medium to strong associations between the factors. These can be explained as illustrated below: Table AF3 shows the intercorrelations between the scales as an indicator of construct validity. The strongest correlation (r= .666**) is found between the factors 1 ‘Insecurity’ and 3 ‘Negative affect & cognitive impairments’. This high correlation seems plausible in terms of the items, as negative emotions are addressed in both areas. The lowest correlation (r= .214**) is found between the factors 2 ‘Balance’ and 4 ‘Worries’. Considering that contrasting constructs are represented here, this correlation looks plausible. All other correlations between these two values are in the medium to strong range.

|  | **1** | **2** | **3** | **4** | **5** |
| --- | --- | --- | --- | --- | --- |
| **1 –** **Insecurity** | 1.000 | .504^**^ | .666^**^ | .588^**^ | .418^**^ |
| **2 – Balance** |  | 1.000 | .373^**^ | .214^**^ | .400^**^ |
| **3 – Negative affect & cognitive impairments** |  |  | 1.000 | .492^**^ | .261^**^ |
| **4 – Worries** |  |  |  | 1.000 | .504^**^ |
| **5 – Manageability** |  |  |  |  | 1.00 |

** Results are significant with p = 0.01 (two-sided).

# Supplemental table 4

**Title:** Descriptive results of the items, (raw, unbanded data; not-imputed data set, N = 223).

| **Item-No.** | **Item** | **applies fully** | **somewhat applies** | **rather not** | **does not apply at all** | **no answer / missing** |
| --- | --- | --- | --- | --- | --- | --- |
|  |  | % (N) | % (N) | % (N) | % (N) | % (N) |
| 1 | I feel detached from others. | 2.2 (5) | 17.0 (38) | 38.1 (85) | 42.2 (94) | 0.4 (1) |
| 2 | I try to hide my *hand* eczema. | 20.2 (45) | 42.2 (94) | 16.6 (37) | 20.6 (46) | 0.4 (1) |
| 3 | I am self-confident. | 30.0 (67) | 43.5 (97) | 20.2 (45) | 5.8 (13) | 0.4 (1) |
| 4 | I am nervous. | 16.6 (37) | 45.3 (101) | 22.9 (51) | 15.2 (34) | - |
| 5 | I envy people with normal skin. | 38.6 (86) | 26.9 (60) | 18.8 (42) | 15.7 (35) | - |
| 6 | I feel I can handle my *hand* eczema. | 3.1 (7) | 33.6 (75) | 42.6 (95) | 20.2 (45) | 0.4 (1) |
| 7 | I feel overwhelmed by my *hand* eczema. | 15.2 (34) | 37.2 (83) | 32.7 (73) | 14.8 (33) | - |
| 8 | I am optimistic about my life with *hand* eczema. | 40.4 (90) | 45.3 (101) | 12.6 (28) | 1.3 (3) | 0.4 (1) |
| 9 | I try to avoid physical contact or touching other people. | 16.1 (36) | 35.0 (78) | 30.9 (69) | 17.9 (40) | - |
| 10 | I struggle with my appearance. | 9.4 (21) | 32.7 (73) | 33.2 (74) | 24.2 (54) | 0.4 (1) |
| 11 | I am a relaxed person. | 21.5 (48) | 47.1 (105) | 25.1 (56) | 5.8 (13) | 0.4 (1) |
| 12 | My *hand* eczema makes me angry. | 14.3 (32) | 40.4 (90) | 25.6 (57) | 19.7 (44) | - |
| 13 | I’m afraid of being rejected. | 9.4 (21) | 18.8 (42) | 42.2 (94) | 29.1 (65) | 0.4 (1) |
| 14 | I am a well-rounded person. | 16.1 (36) | 59.2 (132) | 18.4 (41) | 5.8 (13) | 0.4 (1) |
| 15 | I feel guilty about scratching. | 8.5 (19) | 23.8 (53) | 30.5 (68) | 36.8 (82) | 0.4 (1) |
| 16 | I have difficulties concentrating. | 6.7 (15) | 32.7 (73) | 33.6 (75) | 25.6 (57) | 1.3 (3) |
| 17 | I am in control of my *hand* eczema. | 1.8 (4) | 24.2 (54) | 51.1 (114) | 22.4 (50) | 0.4 (1) |
| 18 | I feel sad about having *hand* eczema. | 27.4 (61) | 44.8 (100) | 15.7 (35) | 12.1 (27) | - |
| 19 | I feel I can do what other people can do. | 15.7 (35) | 40.4 (90) | 29.1 (65) | 14.8 (33) | - |
| 20 | I worry about my life because of my *hand* eczema. | 32.3 (72) | 48.9 (109) | 12.1 (27) | 6.7 (15) | - |
| 21 | I feel embarrassed about my skin appearance. | 19.7 (44) | 30.9 (69) | 31.4 (70) | 17.9 (40) | - |
| 22 | I have no problem with intimacy. | 30.5 (68) | 29.1 (65) | 23.8 (53) | 15.7 (35) | 0.9 (2) |
| 23 | I feel insecure. | 7.6 (17) | 35.0 (78) | 32.7 (73) | 20.6 (46) | 4.0 (9) |
| 24 | I cope well with my *hand* eczema. | 6.7 (15) | 52.5 (117) | 33.6 (75) | 7.2 (16) | - |
| 25 | I feel trapped because of my *hand* eczema. | 22.4 (50) | 51.1 (114) | 16.6 (37) | 9.9 (22) | - |
| 26 | I feel I am good enough as a person. | 20.6 (46) | 49.8 (111) | 22.4 (50) | 5.8 (13) | 1.3 (3) |
| 27 | I’m afraid of being a burden to my relatives. | 6.7 (15) | 16.1 (36) | 30.0 (67) | 46.6 (104) | 0.4 (1) |
| 28 | Itching drives me crazy. | 32.7 (73) | 39.9 (89) | 13.5 (30) | 12.6 (28) | 1.3 (3) |
